# 1. Chronic Bronchitis. 2. Endocarditis

**Published:** 1895-01

**Authors:** R. C. M. Page

**Affiliations:** New York; Professor General Medicine at the New York Polyclinic and Hospital


ATLANTA
### MEDICAL AND SURGICAL JOURNAL.

VOL. XI. JANUARY, 1895. NO. 11.

EDITED BY
LUTHER B. GRANDY, M.D., AND MILLER B. HUTCHINS, M.D.,
WITH THE CO-OPERATION OF
H. V. M. MILLER, M.D., LL.D., VIRGIL O. HARDON, M.D.,
FLOYD W. McRAE, M.D., DUNBAR ROY, M.D.,
AND H. R. SLACK, M.D., PH.M.

### *CLINICAL LECTURE.*

### 1. CHRONIC BRONCHITIS. 2. ENDOCARDITIS.*

BY R. C. M. PAGE, M.D.,
Professor General Medicine at the New York Polyclinic and Hospital.

1. This boy is sixteen years old. He was born in Ireland, and came to this country eleven years ago. For the past two years he has been at work in a carpet factory. He complains of a cough, which he has had for over a year. He tells us that his work confines him to a room where the air is continually laden with cordage filaments, and this at once gives us an idea as to the origin of his cough. Among men who are engaged at certain occupations—miners, carpet-weavers, brush-manufacturers, potters, knife-grinders, brass-finishers, etc.—a form of chronic bronchitis is very commonly seen, to which Zenker has given the name of pneumonokoniosis, which simply means an affection of the lungs arising from the inhalation of dust. In coal-miners it is known as anthracosis pulmonum; in iron-workers it is known as siderosis pulmonum, etc.

* Delivered November 5, 1894.

It is a curious fact that in these cases it is usually the right lung which is affected; this is due to the fact that the right bronchial tube being larger than the left, and the natural continuation of the trachea, the dust enters the right lung much more freely than the left. In a certain percentage of these cases the diseases will eventuate in phthisis, and, hence, we sometimes hear of knife-grinders' consumption, potters' consumption, and the like. There can be no phthisis, however, until the tubercle bacilli attack the patient. Anthracosis or siderosis pulmonum is not the cause of the phthisis, but a powerful predisposing factor in that direction. In many instances the patients simply suffer from a chronic bronchitis. In my experience, the disease is very common among those who work in jute or carpet mills. This young man has been able to throw off the effects of this disease almost as fast as they came on, and he is, as you see, in comparatively good condition. At a more advanced age, say forty or fifty, the probabilities are he would have broken down before this.

Let us now examine this patient. He is pretty well nourished. If the cough from which he has suffered for over a year was due to phthisis, he would probably be more emaciated than he is. The breathing too is not rapid, nor is there any increased frequency of the heart's action. When we ask him to take a deep breath, we see that the expansion on the left side is fuller and quicker than on the other. The right side is evidently crippled, and the left side is doing more than its share of the work. Let us look at some of the diseases which might produce this condition. Restrained breathing on one side may be due to pleurodynia, or muscular rheumatism, as it is known, or intercostal neuralgia; or there may be a more serious condition, such as pleurisy, or old pleuritic adhesions, or pneumonia or phthisis. Let us now pass on to the physical signs. The vocal fremitus on the right side is exaggerated. On percussion, the pitch is higher on the right side than on the left, and the voice sounds are louder. There are no râles. From the boy's history, his appearance, and the physical signs, I am inclined to think that there is peri-bronchial thickening on the right side of the chest. I do not think there is any consolidation of lung tissue. As regards treatment, the first point to insist on is that the patient should find other employment. We cannot cure these cases of mechanical bronchitis unless we remove the cause. Counter-irritation does good in some cases, and for this purpose I know of nothing better than the compound iodine ointment. Besides this the patient should be given some cough mixture to relieve the irritation. For this purpose I usually employ the following, with which you are probably all familiar. It is commonly known as Stokes's expectorant mixture:

℞ Pulv. ammon. carb.......................................gr. xvi.

Ext. scillæ fld.,

Ext. senegæ fld., aa.......................................ʒ ss.

Tin. opii camphoratæ.......................................ʒ iii.

Syr. tolu.......................................℥ .

Aquæ, q. s. ad.......................................℥ ii.

M. Sig.—A teaspoonful every three hours.

2. This boy is eighteen years old; a shirt-maker by occupation. During the past six months he has had a number of attacks of acute articular rheumatism. He comes here complaining of a slight cough and shortness of breath. In patients under the age of twenty who give a history of acute articular rheumatism, we are very apt to find that the disease has been complicated by endocarditis, which has left a valvular lesion behind. The younger the patient, the more likely is it that such a complication will occur. Why this is so we do not know. It is claimed to be due to the fact that as a person grows older, the acidity of the blood diminishes. In these cases of endocarditis, the left side of the heart is usually affected. Endocarditis occurring during fetal life usually affects the right side of the heart, which is supposed to be due to the fact that during that period the right side of the heart has a great deal more to do than the left. Endocarditis may result in one of three ways: It may end in resolution, leaving no trace behind, which is a very fortunate mode of termination for the patient; or, secondly, it leaves a valvular lesion; or, lastly, and this is rather rare, it will leave a lesion which is situated somewhere within the ventricles, which is known as intra-ventricular lesion, in contradistinction to a valvular lesion. How do we tell these two apart? With a valvular lesion, not only the valves but also the orifices may be involved. There may be insufficiency of the valves and constriction of the orifice at the same time, so that obstruction and regurgitation may exist together at the same orifice, and this occurs more frequently than we suppose. In fact, I am inclined to believe that mitral regurgitation is nearly or always associated with mitral obstruction. An intra-ventricular lesion has nothing whatever to do with the valves or orifices. It may be a roughened spot on the wall of the ventricle, or it may be a fibrinous thread left there, or a twisting of one or more of the columnæ carneæ. Of the valvular lesions met with, mitral regurgitation is the most common. Next to that, in my opinion, is mitral obstruction. If the lesion in this case is a mitral one, then its murmur should be heard at the apex, if it can be heard anywhere, inasmuch as the apex is formed of the left ventricle. If the murmur be that of mitral regurgitation, we shall hear it not only at the apex, but also behind, between the lower angle of the scapula and the spine, and we can in this manner distinguish it from intra-ventricular murmur, because the latter is never heard behind. Furthermore, with a mitral regurgitant murmur the heart is usually enlarged, while when the lesion is intra-ventricular, it is not enlarged. In this case there is a very distinct blowing murmur which is heard at the apex and conveyed to the left; it is also plainly heard behind. His attacks of rheumatism are of such recent date that the heart has not yet had time to enlarge very much. How can we distinguish a mitral regurgitant murmur from one due to mitral obstruction? With either lesion, unless it is of recent date, there is more or less cardiac enlargement. The difference lies in the fact that with mitral regurgitation you get enlargement of the left ventricle, while with mitral obstruction alone you do not get enlargement of the left ventricle; in fact, you get rather an atrophy of the left ventricle. Furthermore, with mitral obstruction you would not get the murmur behind, and the apex-beat of the heart would be very feeble. The right ventricle being situated in front of the left, and the latter being smaller than it is in health, it would be crowded away from the chest wall, and the impulse of the apex-beat would thus be lessened. With mitral regurgitation, on the contrary, we would look for an increased apex impulse.

Among the laity the dread of sudden death from heart disease is very general. As a matter of fact, sudden death from this cause is rather uncommon. Let us look at some of the conditions which may produce it: (1) Aortic regurgitation. With this form of cardiac disease, death is sure to result sooner or later, because the disease is progressively hopeless. I have never seen any such patients improve much under any form of treatment excepting those in whom the disease was dependent on syphilis. With this form of cardiac trouble the patient not only grows gradually worse, but he may drop dead at any moment, and the mode of this death has never been satisfactorily explained. It seems to me that the best explanation that can be given is that the left ventricle becomes more and more enlarged, until we get what is known as the *cor bovis*, or ox heart, and in some of these cases there will be pressure on the coronary vessels which feed the heart; the patient then dies of anemia of the heart, very much as it occurs in angina pectoris. In fact, aortic regurgitation is not infrequently associated with attacks of angina, due to this pressure on the coronary vessels. (2) Angina pectoris. A patient with this affection may die in the first attack, or he may survive a dozen or more attacks. With the more recent methods of treatment, the mortality from this disease has been considerably reduced. (3) Fatty heart. By this I mean a fatty metamorphosis of the muscular structures of the heart. This process may go on until the time comes when the heart simply ceases to beat. (4) Cardiac aneurism. In these cases the ventricular wall becomes thinner and thinner until rupture and sudden death occur.

The above are about the only conditions of the heart in which sudden death is likely to occur. All valvular lesions will produce death in time, but the termination will not be of sudden onset and will usually be the result of some complication. With mitral lesions the patients do not die suddenly. They usually die from some complication, notably hemorrhagic infarction of the lungs, pneumonia, etc. In either mitral obstruction or regurgitation, we are sure to have enlargement of the right ventricle, which gradually becomes weakened and the circulation becomes sluggish; thrombi then form about the trabeculæ, and from these emboli are carried into the lungs through the pulmonary artery. They occlude the smaller pulmonary vessels, and a hemorrhagic infarction is the result. These areas of infarction may be multiple. In addition, with a mitral lesion, the lungs being always more or less engorged, the blood is thrown back upon the inferior vena cava, producing congestion of the stomach, liver, and bowels; in the liver this condition produces what is known as the nutmeg liver, or red cirrhosis; in the stomach it produces chronic gastritis, and in the bowels intestinal catarrh.

Of the two, mitral obstruction is more fatal than mitral regurgitation, yet these patients often reach the age of fifty or sixty, or over. Let us look at the character of the pulse in these various cardiac affections. An intermittent pulse does not necessarily indicate a valvular lesion; it is more significant of some reflex cardiac disturbance, and is often the result of dyspepsia. Irregular heart action may be due (1) to centric causes, as we see it in chorea, epilepsy, hysteria, or spinal irritation, especially if they are attended by insomnia. Also in persons who study very hard. (2) It may be the result of reflex causes, as we se it in dyspepsia, or after the ingestion of certain articles of food, or from the use of tobacco. The so-called "tobacco heart" is nothing more than cardiac irritability brought about by the action of tobacco on the ganglia of the heart. The abuse of tea or coffee may produce the same effect. (3) Blood changes. In certain diseases, such as rheumatism, Bright's disease, anema, etc., the blood may be in such a vitiated condition as to produce irregular heart action without any actual disease of the latter organ. (4) Mechanical causes. Under this head may be included all conditions where there is obstruction to the pulmonary circulation; displacement from various tumors, effusions and the like. Tight lacing is a very common cause of irregularity of the heart.

